# Dolphin Longevity and Long‐Term Site Fidelity in the Mississippi Sound: Insights From a 1982 Health Assessment

**DOI:** 10.1002/ece3.74432

**Published:** 2026-09-28

**Authors:** Moby Solangi, Forrest Collins, Kailey Pamperin, Theresa Madrigal, Kelsi Busboom

**Affiliations:** ^1^ Institute for Marine Mammal Studies Gulfport Mississippi USA

**Keywords:** Gulf, longevity, photo‐ID, site fidelity, stock, *Tursiops truncatus*

## Abstract

Due to its stable and safe environment, The Mississippi Sound (MSS) is home to one of the densest populations of common bottlenose dolphin ( *Tursiops truncatus* ) in the Gulf of Mexico. Being apex predators and sentinel species, the bottlenose dolphin acts as an ecological indicator within the MSS. In 1982, a health assessment was initiated to monitor the health of the dolphins and the MSS. Individuals were freeze branded and tracked in a long‐term mark‐tracking study. To monitor these individuals after the study, the Institute for Marine Mammal Studies (IMMS) conducted photo identification surveys to observe the dolphin population within the MSS. From the 50 dolphins originally freeze‐branded, 10 have been observed from 2011 to 2026. Seven are currently over 30 years old, and one is confirmed to be 47 as of 2026. Additionally, of the seven live dolphins, two have shown evidence of long‐term site fidelity. As some of the 1982 dolphins have confirmed re‐sightings 29 years after the initial freeze branding, it is vital to continue this monitoring effort through photo identification to accurately record dolphin longevity, long‐term site fidelity, and other dolphins that may be resident or transient. However, given the limitations of mark‐tracking studies, continued efforts are needed to confirm site fidelity and longevity. Ultimately, this study aims to create a better understanding of not only the dolphin's health in the MSS, but also the health of the ecosystem they live in.

## Graphical abstract

Current photo‐ID surveys have identified 10 common bottlenose dolphins that were originally freeze‐branded from a health assessment in the Mississippi Sound from 1982. These dolphins have experienced long‐term site fidelity with confirmed ages being above 30+ years old, and one individual being 47 years old. With longevity and long‐term site fidelity being highly debated concepts in dolphin life history, the continued monitoring of this regional population is vital in conserving the species.

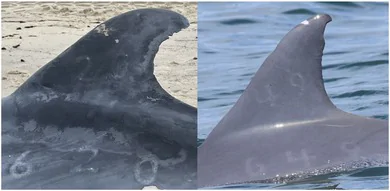

## 1 Introduction

The Mississippi Sound (MSS) represents vital foraging, reproduction, and calving grounds for one of the densest populations of bottlenose dolphins, *Tursiops truncatus* (also known as “bottlenose dolphin(s)” throughout this article) in the Gulf of Mexico due to its relatively stable environment and for the resources (both abiotic and biotic) it provides (Pitchford et al. 2016). This population falls within the Northern Gulf of Mexico Bay, Sound, and Estuary Stock (Hayes et al. 2023). The MSS is a unique estuarine ecosystem in the northern Gulf of Mexico with complex food webs (Cloyed et al. 2021) and relatively stable bottlenose dolphin movements (Pitchford et al. 2016), with evidence of migration during the winter months offshore into deeper waters following prey movement (Miller et al. 2013) with return in the spring and summer months for calving (Suffoletta et al. 2026). This population observed in this area is a mix of resident and transient individuals (Hubard et al. 2004; Mullin et al. 2017; Pamperin et al. 2025; Giordano et al. 2026).

Bottlenose dolphins are known to reside in temperate and tropical waters within coastal and bay areas (Caldwell and Caldwell 1972; Cloyed et al. 2021). Within the MSS, bottlenose dolphins have shown to exhibit site fidelity, with multiple individuals being observed returning to select habitats (Pamperin et al. 2025). Additionally, these bottlenose dolphins represent apex predators within their ecosystem and shift their ecology based on the changes they experience within their environment (Wells et al. 2004). Due to their behavior and impact on the food web within the MSS, bottlenose dolphins act as sentinel species within their ecosystem as their ecology, life history, and behavior will reflect environmental changes (Wells et al. 2004). The MSS has historically been a region that has been impacted by natural and anthropogenic events, which ultimately have affected the ecosystem (Abbot‐Jamieson and Ingles 2015; Stacy et al. 2019). As such, monitoring bottlenose dolphins within the MSS is vital to better understanding the health of the species and the health of the ecosystem.

Being a sentinel species, it has become paramount to determine the age of bottlenose dolphins to better understand how changes within their ecosystem can better be monitored. However, different methods and studies have suggested varying bottlenose dolphin longevity estimates within the Gulf of Mexico. One study, conducted in the eastern Gulf of Mexico, estimates life span to be less than 30 years old (Odell and Schneyer 1983), while other studies across the Gulf and the Atlantic coast have indicated that longevity can span beyond 40 years old with rare occurrences past 50 years old (Hohn et al. 1989; Fernandez and Hohn 1998; Wells and Scott 1999; Barratclough et al. 2024). Methods such as tooth‐cross sectioning (Hohn and Fernandez 1999) and skin epigenetic DNA methylation (Barratclough et al. 2024) have been used to estimate age.

However, to accurately estimate longevity within a species, it requires a long‐term mark‐tracking study to monitor and observe select individuals within the population. One such method of doing this is “freeze‐branding”. Freeze‐branding is a method that has been used successfully in many cetacean studies in the past, before advancements in photographic technology that have led to present day computer‐assisted fin recognition (Evans et al. 1972; Solangi and Dukes 1983; Fair et al. 2006; Thompson et al. 2019). Long exposure freeze‐branding is a technique that changes both the pigment cells and hair follicles (Melin et al. 2006), leading to longer‐lasting brands. Although most brands fade to some capacity with time, brands that have faded can still be identified in later years through remaining partial numbers (Boyd et al. 2010). Freeze‐branding in bottlenose dolphins is applied to the body and dorsal fin to help identify dolphins later through research, like photo‐identification (photo‐ID) (Fair et al. 2006). Freeze‐branding is still a method used with programs that consistently perform health assessments and information from these studies can provide insight into age estimation and location patterns, like site fidelity, of these individuals (Fair et al. 2006).

The objectives of this study are to provide longevity estimates and long‐term site fidelity evidence of select bottlenose dolphins that were freeze branded in Solangi and Dukes (1983) final report (also known as the “health assessment”). Bottlenose dolphin longevity is still a highly debated topic due to possible regional (Danil and Chivers 2007), morphometric (Wells and Scott 1999), molecular (Barratclough et al. 2024), and sex differences (McEntee et al. 2023), and the freeze‐branded individuals allow for greater insight into the observed ages bottlenose dolphins can reach. Additionally, as these bottlenose dolphins are so uniquely marked, the ability to track and obtain location data through photo‐ID work provides a unique opportunity to observe site fidelity more than 40 years after the health assessment was performed. Ultimately, addressing and estimating the age of these bottlenose dolphins while noting resident dolphins within the Mississippi Sound will provide a better understanding into the behavior, ecology, and population structure of bottlenose dolphins in the northern Gulf of Mexico.

## 2 Methods

### 2.1 MSS Health Assessment 1982

A health assessment of the bottlenose dolphins, *Tursiops truncatus*, in the MSS was performed from the 28 of June to the 2 of August 1982 (Solangi and Dukes 1983). This study was conducted within the Mississippi Sound, off the coast of Mississippi, United States of America (from 30.177046°, −89.393116° to 30.260650, −88.389646°). During this study, a combination of four boats and an observational aircraft was used to search for bottlenose dolphin groups. The crew included experienced divers, boat captains, a pilot, principal investigators, and representatives from Animal and Plant Health Inspection Service (APHIS) and National Marine Fisheries Service (NMFS).

Once a group of bottlenose dolphins was sighted, one boat evaluated the number and size of individuals within that group to determine that there were no nursing calves, and the group size was no more than five animals. Two boats worked together to drop a net (457 m × 5 m net with a mesh size of 15 cm) in a clockwise direction to capture the bottlenose dolphins. The circumference of the net was tightened until a diver could respond to help support the individual(s) within the net. Once in the net, a diver kept the animal(s) above water until the boat conducting morphometrics and other testing (described below) approached. Once an individual(s) was captured, this was considered a “capture event.” The location, number of bottlenose dolphins, individuals processed and released, processing time, and environmental conditions were all recorded for each capture event. This protocol was adapted and adjusted from similar health assessments performed around this time (Asper 1975; Odell and Asper 1982; Odell and Schneyer 1983).

Each individual captured was given numerical liquid‐nitrogen‐dipped marks (“freeze‐brand”) starting from 601 to 653 (the number 6 indicated the MSS, while the last two digits indicated the individual). Two digits (01‐53) were placed on each side of the dorsal fin (to indicate the individual by boat or aerial surveys), and all three digits were placed on both sides of the animal, below the dorsal fin (if the dorsal fin was absent) (Figure 1). Along with freeze‐branding each individual, morphometrics (sex, weight, lengths), blood (for hematology, chemistry, endocrinology, and biochemical genetics), cultures (from skin, blubber, and liver biopsies), and a picture of both the dorsal fin and fluke were taken (Solangi and Dukes 1983).

**FIGURE 1:**
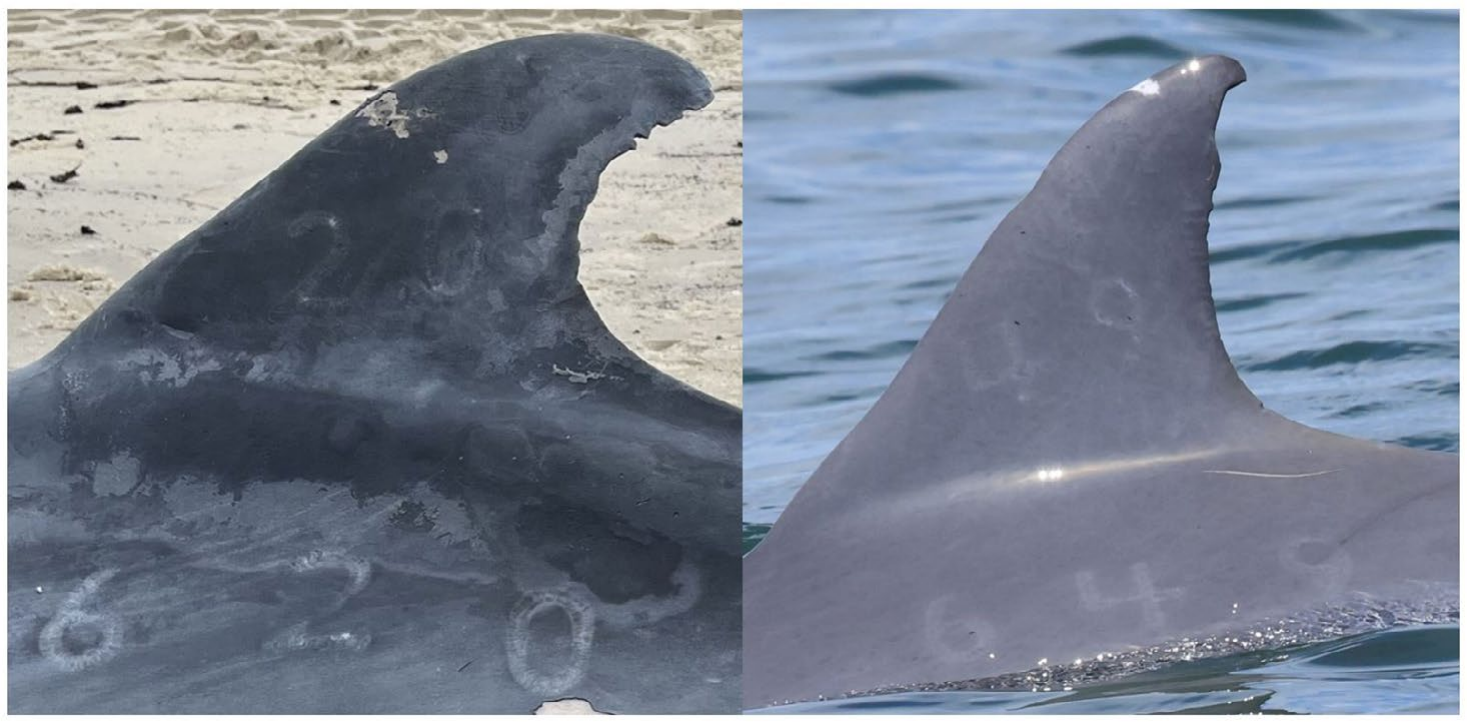
Image of two freeze‐branded individuals from Solangi and Dukes (1983). F620 (left) was reported deceased in 2017. F649 (right) was last seen through photo‐ID in June 2026.

Tooth extractions and age estimations were conducted by Odell and Schneyer (1983) as part of the 1982 health assessment. This was done by removing one maxillary tooth from the lower jaw of the individual. Once extracted, a tooth was boiled in tap water for 5 min before scraping off the adhering tissue. The teeth were then stored in a 50:50 mixture of 70% ethanol and glycerol with a few crystals of thymol to prevent fungal growth. Two methods were used to estimate the age of an individual: thin section (T‐section) and etch. The T‐section was taken from the center of each halved tooth. Once the T‐section was completed, the half‐tooth was etched. Growth layer group counts from both methods were compared. Age estimates from both methods generally matched, but in instances where they did not, the higher age estimate was used.

### 2.2 Estimated Longevity

From 1982 to 2009, a community‐driven bottlenose dolphin observation protocol was initiated so the community could report sightings of these freeze‐branded individuals. As reports of these branded individuals declined into the early 2000s, the Institute for Marine Mammal Studies began bottlenose dolphin monitoring and research in the MSS through photo‐ID surveys starting in 2010. Photo‐ID followed methodologies found in Pamperin et al. (2025). Freeze‐brands were confirmed to be from the 1982 study if there were two numbers on each side of the dorsal fin ranging from 01 to 53, and/or there were three numbers on each side of the body ranging from 601 to 653. Currently in the MSS, the Solangi and Dukes (1983) and the Mullin et al. (2017) were the only animals present with freeze‐branded numbers. However, there have been other freeze‐branding studies since the Solangi and Dukes (1983) study within the Gulf (such as Texas and Florida) that, in the event of large‐scale movements, would not conflict with our observations for the current study (Wursig and Lynn 1996; Fair et al. 2006).

To determine if any bottlenose dolphins with freeze‐branding from the 1982 health assessment stranded, stranding data from IMMS and regional records from 2000 to 2026 was examined. If a freeze‐brand was found, there was confirmation through pictures and “Level A” data (morphology, life history, biology, and general health) to confirm that it was from the 1982 health assessment (Examiners Guide 2021). Data collected on these bottlenose dolphins included date and location of stranding, as well as sex, length, and weight of the individual (if body condition allowed for the measurement).

The current age at the time of observation was estimated for both live and stranded bottlenose dolphins. For the individuals seen through photo‐ID, their age at last confirmed sighting was estimated. For the stranded bottlenose dolphins, the age at time of death was calculated. Alongside age, the GPS location of the 1982 freeze‐branded bottlenose dolphin was recorded per individual. The GPS points (decimal degrees) of the last known photo‐ID sighting and stranding location were also recorded. These points were displayed on a map using *ArcGIS Pro* 3.1.2.

### 2.3 Site Fidelity

Site fidelity is the likelihood of an individual to remain in or return to a given area (Mintzer et al. 2023). Bottlenose dolphins in the MSS (within the waters of Mississippi from the border of Louisiana to the border of Alabama) are considered to have site fidelity to the area if they have been seen five times or more (Pamperin et al. 2025). Since photo‐ID surveys began in 2010, the number of sightings for each freeze‐branded individual was recorded to determine if they had site fidelity to the MSS. The freeze‐branded individuals that did have site fidelity were displayed on a map through *ArcGIS Pro* 3.1.2 using their sighting points.

For the freeze‐branded individuals with site fidelity, additional descriptors of resighting histories from the on‐going photo‐ID study (August 1, 2010, to June 30, 2026) were calculated. This included permanence, periodicity, and temporal patterns. Permanence, or duration, is the time from the first to last sighting of an individual, and periodicity is the recapture frequency (Tschopp et al. 2018). Permanence was calculated by dividing the number of months from the first to last sighting of an individual by the number of months surveyed during the study period. Periodicity was calculated by dividing the number of months an individual was sighted in by the number of months from their first to last sighting (Pamperin et al. 2025). Both permanence and periodicity result in values between 0 and 1. For temporal patterns, months were split into winter (December–February), spring (March–May), summer (June–August), and fall (September–November) (Pamperin et al. 2025).

## 3 Results

### 3.1 Estimated Longevity

A total of 50 bottlenose dolphins was captured and freeze‐branded to assess longevity (Solangi and Dukes 1983). Of those 50 bottlenose dolphins, 10 (seven live and three dead) have been observed and recorded using photo‐ID and stranding data since 2001 (Table 1). No age estimates were gathered in 1982 for F623, so F623 was not included with longevity analysis. Of the remaining nine bottlenose dolphins, seven individuals were all above the age of 30 years old at their last confirmed sighting, with the oldest confirmed age at 47 years old (F649) (Table 1). Of the two that were confirmed deceased, one had the youngest confirmed age from the study (F632) at 27 years old (Table 1).

**TABLE 1:** List of freeze‐branded dolphins observed (either by photo‐ID or stranding) after the 1982 study.

Number	Freeze brand number	Date marked dd/mm/yyyy	Current status	Confirmed status date dd/mm/yyyy	Latitude marked	Longitude marked	Sex	1982 age	Confirmed age
1	F603	28/06/1982	Live	06/08/2020	30.238889	−89.158333	M	3	41
2	F606	28/06/1982	Live	01/06/2011	30.3208333	−89.1013889	F	5	34
3	F608	30/06/1982	Live	03/08/2015	30.23861	−89.0833333	M	9	42
4	F625	07/07/1982	Live	29/07/2013	30.354167	−88.86667	F	3	34
5	F626	13/07/1982	Live	04/08/2011	30.339167	−89.116944	F	2	31
6	F642	27/07/1982	Live	15/05/2012	30.293056	−88.5791667	M	7	37
7	F649	02/08/1982	Live	02/06/2026	30.2552778	−89.058611	F	3	47
8	F620	07/07/1982	Dead	11/01/2023	30.3013889	−89.1775	F	4	45
9	F623	07/07/1982	Dead	03/08/2001	30.313333	−89.188889	F	NA	NA
10	F632	20/07/1982	Dead	23/01/2002	30.339167	−89.116944	F	7	27

### 3.2 Site Fidelity

Since 2010 (when IMMS began photo‐ID surveys), two out of the seven bottlenose dolphins that were last observed to be alive through photo‐ID displayed site fidelity to the MSS. Dolphin F603 was freeze‐branded on the western tip of Cat Island (Figure 2) and was seen six times from October 2012 to August 2020, exclusively around Cat Island, confirming site fidelity to that area (Table 2, Figure 3). Over the course of the 6 sightings, he was associated with 9 individuals that also displayed site fidelity. F603 was sighted over a course of 7.83 years with an average of 15.74 months between sightings. The smallest time between two sightings was 16 days, and the longest was 3.93 years. F603 had a permanence value of 0.50 and a periodicity value of 0.06. Temporally, he was sighted 3 times in the summer and 3 times in the fall.

**FIGURE 2:**
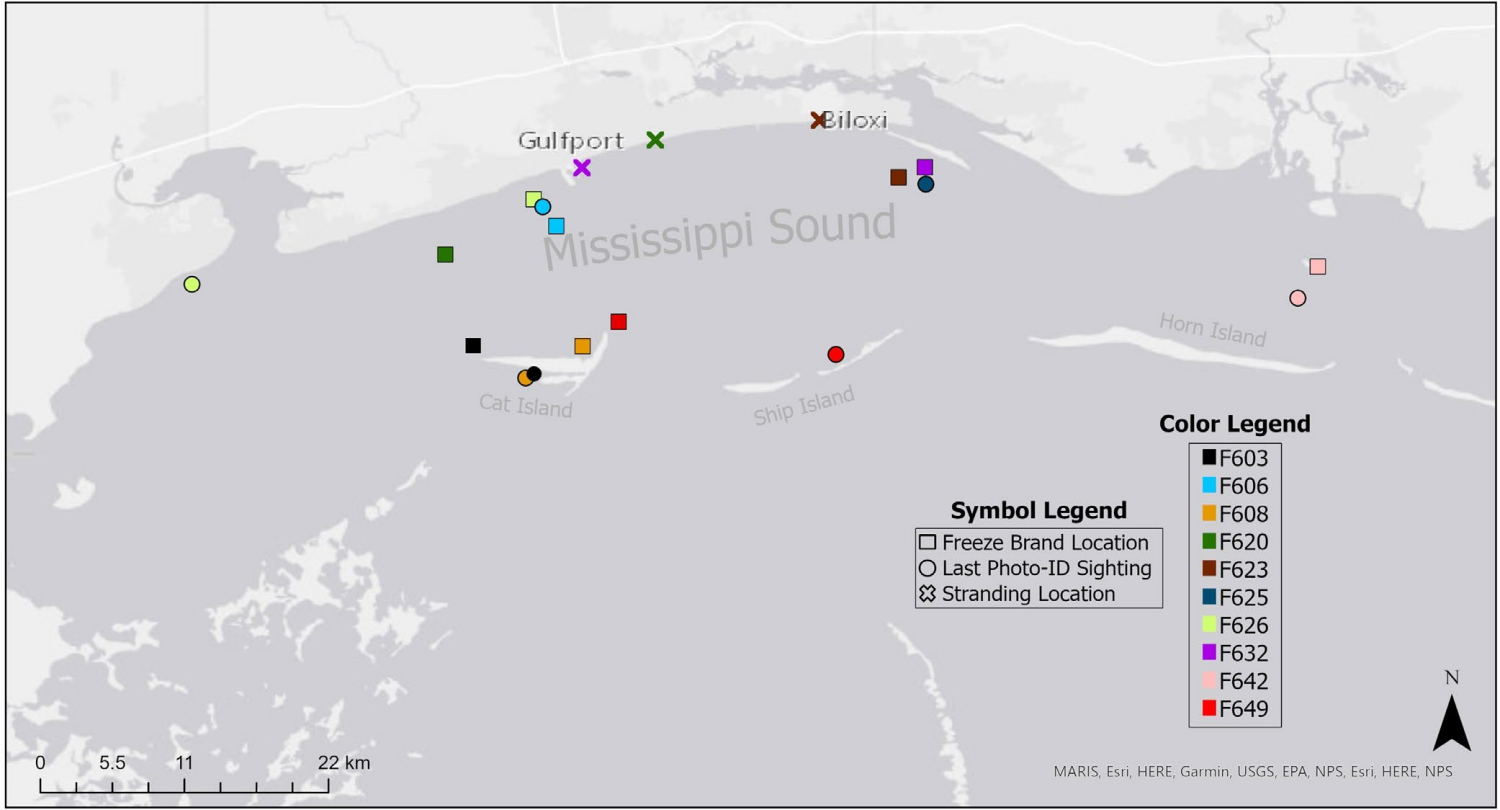
Freeze‐brand locations from the 1982 study along with last confirmed sighting points (photo‐ID or stranding). Please see Table 1 for confirmed sighting point data.

**TABLE 2:** Photo‐ID sighting timeline for F603 and F649.

F603			F649		
Sighting number	Date (dd/mm/yyyy)	Season	Sighting number	Date (dd/mm/yyyy)	Season
1	10/10/2012	Fall	1	10/06/2015	Summer
2	03/08/2015	Summer	2	07/06/2016	Summer
3	07/10/2015	Fall	3	09/03/2017	Spring
4	17/08/2016	Summer	4	09/05/2017	Spring
5	02/09/2016	Fall	5	25/04/2018	Spring
6	06/08/2020	Summer	6	15/08/2019	Summer
			7	04/03/2022	Spring
			8	04/05/2022	Spring
			9	20/09/2022	Fall
			10	18/04/2023	Spring
			11	23/06/2023	Summer
			12	23/08/2023	Summer
			13	20/03/2024	Spring
			14	20/05/2024	Spring
			15	12/07/2025	Summer
			16	02/06/2026	Summer

**FIGURE 3:**
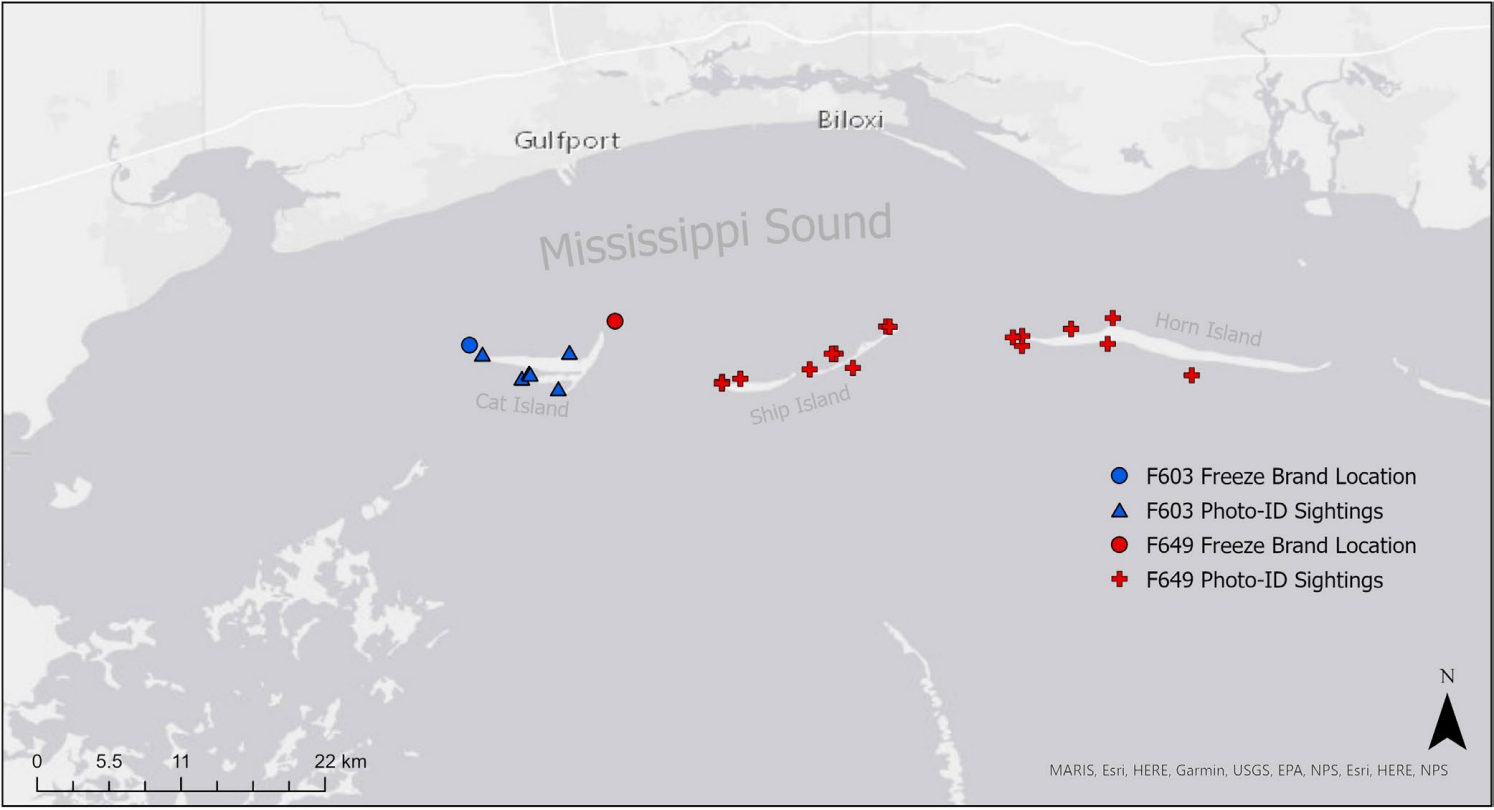
Two freeze‐branded dolphins that displayed site fidelity to the Mississippi Sound from 2010 to 2026.

The second bottlenose dolphin, F649, was freeze‐branded on the northern tip of Cat Island (Figure 2). F649 has been seen 16 times from June 2015 to June 2026 (Table 2). Over the course of the 16 sightings, she had associated with 63 individuals that also had site fidelity to the MSS. She was seen over a course of 10.98 years with an average of 8.79 months between sightings. The shortest time between sightings was 2.00 months and the longest was 2.55 years. This individual has been sighted around Ship and Horn Islands (Figure 3), showing a preference for the islands within the MSS. F649 had a permanence value of 0.69 and a periodicity value of 0.12. She was sighted 8 times in the spring, 7 times in the summer, and once in the fall.

## 4 Discussion

The 1982 health assessment was an essential tool to better understand bottlenose dolphin longevity in this region and long‐term site fidelity to the MSS. Of the 50 bottlenose dolphins that were originally freeze‐branded, IMMS was able to observe and monitor 10 of them. Although this is only 20% of the bottlenose dolphins branded during the 1982 health assessment, the photo‐ID performed by IMMS further complements the health assessment. Additionally, with the combination of multiple datasets (such as the 1982 health assessment, historical community‐driven observations, current IMMS photo‐ID surveys, and regional stranding records), the data originally collected by the 1982 health assessment can be used to enhance conservation and management strategies to further protect the dolphin population in the MSS.

Using the current IMMS photo‐ID surveys, seven of the 10 bottlenose dolphins were confirmed alive past 2011 (Table 1). Most freeze‐branded bottlenose dolphin sightings occurred at the conception of the photo‐ID surveys in the early 2010s (2011–2015) with multiple sightings before the last confirmed status date (Table 1). However, as time has progressed, fewer sightings have occurred to the point that only one individual (F649) is currently and regularly being sighted around Ship Island (Table 1, Figures 2 and 3). Due to F649's regular sighting, we were able to confirm long‐term habitat usage and site fidelity alongside longevity estimates (Table 1). Corresponding to their last status confirmation, of these 10 individuals observed throughout the study, 8 were over 30 years old (Table 1, Figure 2), with F649 being confirmed 47 years old in 2026. However, for individuals that have not been confirmed deceased paired with the long and irregular intervals between photo‐ID sightings, four individuals (F603, F606, F625, F626) would also be projected to be over 40 years old if sighted in 2026, and two individuals (F608 and F642) would be projected to be over 50 years old. Lack of regular sightings could also be due to migration rates in and out of the MSS. This result is supported by other studies that have observed other Gulf bottlenose dolphin populations being around and above 40 years old with some evidence being above 50 years old (Hohn et al. 1989; Fernandez and Hohn 1998; Wells and Scott 1999; Barratclough et al. 2024).

Of the other three bottlenose dolphins (F620, F623, and F632) that were confirmed deceased, F623 was the only dolphin for which initial and final age estimates were not recorded, although a tooth was collected at necropsy for future age estimations. F620 had a confirmed age of 45 years at death (Table 1). Although her death was neither confirmed nor denied to be age‐related, natural, or unnatural (i.e., human interference) when she was found stranded, this supports the hypothesis that bottlenose dolphins within the Gulf of Mexico have a longevity greater than 40 years old (Hohn et al. 1989; Fernandez and Hohn 1998; Wells and Scott 1999; Barratclough et al. 2024). Likewise, F632 was found stranded at the age of 27, however, the cause of her death was neither confirmed nor denied to be age‐related, natural, or unnatural (i.e., human interference).

There is strong evidence that the freeze‐branded individuals not only had site fidelity to the MSS, but also long‐term site fidelity. Combining the sightings points from the 1982 study with photo‐ID data shows these individuals often return to the MSS, supported by the permanence and periodicity values, and temporal patterns. The strongest evidence for this is F649. She was seen once during the observational period of the 1982 study (Solangi and Dukes 1983), once during a study from May 1995 to September 1996 (Hubard et al. 2004), and 16 times during the ongoing photo‐ID study (2010 to present). Most site fidelity studies only cover a period of a few years (Bouveroux et al. 2014; Passadore et al. 2018; Takeshita et al. 2021), but this study shows evidence of long‐term site fidelity spanning nearly half a century.

Further looking at sighting patterns for F603 and F649, F603 was seen in the summer and fall months and F649 was seen mostly during the spring and summer months. This type of sighting pattern aligns with previous studies of site fidelity and seasonal movements and migration in and out of this area (Hubard et al. 2004; Mullin et al. 2017), and in addition to displaying long‐term site fidelity, both F603 and F649 would be classified as seasonal residents to the MSS (Mullin et al. 2017). When F603 and F649 were sighted around the barrier islands, they ranged near the islands (traveling between the north and south sides). This type of ranging pattern and behavior has been previously observed in the Mississippi Sound (Mullin et al. 2017). The lack of sightings during the cold months indicates that not only these two individuals, but a portion of individuals sighting within the MSS, are likely migrating offshore to deeper water during those months, potentially following the movement of prey species, then migrating back to the MSS during the spring months (Hubard et al. 2004; Miller et al. 2013), supporting migration rates in and out of the MSS. This movement outside the MSS suggest this population ranges past the currently defined stock boundaries (Giordano et al. 2026), supported by previously found genetic similarities between individuals within the MSS and individuals further in adjacent waters (Vollmer et al. 2021).

As IMMS continues the photo‐ID study, more data will be obtained to better understand bottlenose dolphin longevity and long‐term site fidelity. As seen within this study, time since last sighting can span from a few months (F649 last confirmed sighting: 2026) to many years (F606 and F626 last confirmed sightings: 2011; 29+ years), due to the low probability of detection due to the large population and large study area. This range of photo‐ID creates the possibility that more bottlenose dolphins from the 1982 health assessment can possibly be observed, recorded, and monitored as time progresses. Being sentinel species, the bottlenose dolphins within the MSS have been significantly affected by natural events (such as freshwater inputs from weather events), anthropogenic events (such as freshwater diversions and the Deepwater Horizon Oil Spill), and human interactions (such as recreational and commercial fisheries), which have ultimately reflected changes within their ecosystem (Abbot‐Jamieson and Ingles 2015; Stacy et al. 2019). As the bottlenose dolphin population has been shown to rebound from such environmental effects, continuing to survey these animals within the region will allow us to better understand the different populations and the overall health of the MSS (Suffoletta et al. 2026).

The current study focused on the MSS population, but there are other photo‐ID networks throughout the Gulf of Mexico. As IMMS is not a member of these other networks, future studies would benefit from collaboration with these networks. Freeze‐branding, historically, has been a means to mark and track bottlenose dolphins and other species (Irvine and Scott 1984; Harkonen et al. 1999; Sherwin et al. 2002). However, there are better means to mark, track, and define individuals through modern technologies and techniques (i.e., acoustic and satellite telemetry, stable isotopes, and eDNA) to avoid stress and pain on the animal (Mullin et al. 2017; Hohn et al. 2017; Alter et al. 2022). Additionally, most of our results relied on photo‐ID surveys and stranding records to determine longevity and long‐term site fidelity. However, these methods are not without their associated limitations such as time (such as when a survey or stranding occurs), seasonality (low and peak stranding seasons and dolphin densities), location (of said survey or stranding), weather, and public response (reports of a stranding). Additionally, these irregular intervals between sightings and relying on public response/observation in strandings could lead to survival‐bias observed within the study. Although only 20% of the original freeze‐branded individuals have been sighted, the probability of missing these dolphins during a survey or at a different survey route, and/or a deceased dolphin not being reported due to carcass drift, sinking, or no public response is possible. As these are long‐term data sets, we had the benefit of circumnavigating sporadic absences of photo‐ID surveys throughout the years (2010 to present). However, due to natural currents, climate events, movements and migration, and human interactions (such as commercial and recreational fisheries), we could not fully assume longevity or site fidelity on dolphins that were not regularly sighted and/or stranded.

As bottlenose dolphins are sentinel species, both their longevity and site fidelity are important factors that can indicate the health of the ecosystem (Takeshita et al. 2021; Barratclough et al. 2024; Pamperin et al. 2025; Rouby et al. 2025). Evidence of long‐lived individuals shows that these dolphins interact with their environments across decades and can reflect long‐term trends in ecosystem health. Being a sentinel species, any natural and unnatural (such as anthropogenic events) changes the MSS experiences will directly affect the bottlenose dolphins in the region. In turn, if there is degradation of these individuals' habitat, it will influence their overall longevity and site fidelity toward a specific area, ultimately affecting the biology, ecology, and life history of these animals. The health assessment was a useful tool to determine bottlenose dolphin population health in 1982, and insights are still being gathered from observing the freeze‐branded individuals today. Future observations will be vital in determining the health of the bottlenose dolphin population and the MSS, but this will require long‐term and site‐specific management strategies and conservation efforts that protect the bottlenose dolphin population in the MSS.

## Author Contributions

**Moby Solangi:** conceptualization (equal), data curation (equal), resources (equal), supervision (equal), writing – review and editing (equal). **Forrest Collins:** formal analysis (equal), investigation (equal), methodology (equal), project administration (equal), supervision (equal), validation (equal), writing – original draft (equal). **Kailey Pamperin:** data curation (equal), formal analysis (equal), investigation (equal), validation (equal), writing – original draft (equal). **Theresa Madrigal:** data curation (equal), writing – review and editing (equal). **Kelsi Busboom:** data curation (equal), writing – review and editing (equal).

## Funding

This work was supported by the Mississippi Department of Marine Resources (311500.01, 313021, S‐12‐IMMS‐EDRP1‐10), National Marine Fisheries Service, NA82‐GA‐C‐00023.

## Conflicts of Interest

Dr. Moby Solangi would like to declare a conflicts of interest with the Mississippi Department of Marine Resources, as he is the holder of grants from this institution.

## Data Availability

Data sharing not applicable—no new data generated, or the article describes entirely theoretical research. All data in the current manuscript is reported within the manuscript.

## References

[ece374432-bib-0001] Abbot‐Jamieson, S. , and P. Ingles . 2015. “Natural and Manmade Disasters: Continuing Nemesis for Louisiana, Mississippi, and Alabama Fishing Communities Pre‐ and Post‐Katrina.” Marine Fisheries Review 77: 1–16.

[ece374432-bib-0002] Alter, S. , C. King , E. Chou , S. Chin , M. Rekdahl , and H. Rosenbaum . 2022. “Using Environmental DNA to Detect Whales and Dolphins in the New York Bight.” Frontiers in Conservation Science 3: 820377.

[ece374432-bib-0003] Asper, D. 1975. “Techniques of Live Capture of Smaller Cetacea.” Journal of the Fisheries Research Board of Canada 32: 1191–1196.

[ece374432-bib-0004] Barratclough, A. , R. Takeshita , L. Thomas , et al. 2024. “Estimating Age and Investigating Epigenetic Changes Related to Health Across Multiple Bottlenose Dolphin Populations.” Biological Conservation 293: 110570.

[ece374432-bib-0005] Bouveroux, T. , J. Waggitt , A. Belhadjer , P. Cazenave , P. Evans , and J. Kiszka . 2014. “Modelling Fine‐Scale Distribution and Relative Abundance of Harbour Porpoises in the Southern Bight of the North Sea Using Platform‐Of‐Opportunity Data.” Journal of the Marine Biological Association of the United Kingdom 100: 481–489.

[ece374432-bib-0006] Boyd, I. , W. Bowen , and S. Iverson . 2010. Marine Mammal Ecology and Conservation: A Handbook of Techniques. Oxford University Press.

[ece374432-bib-0007] Caldwell, D. , and M. Caldwell . 1972. The World of the Bottlenose Dolphin. Lippincott.

[ece374432-bib-0008] Cloyed, C. , B. Balmer , L. Schwacke , et al. 2021. “Interaction Between Dietary and Habitat Niche Breadth Influences Cetacean Vulnerability to Environmental Disturbance.” Ecosphere 12: e03759.

[ece374432-bib-0009] Danil, K. , and S. Chivers . 2007. “Growth and Reproduction of Female Short‐Beaked Common Dolphins, *Delphinus delphis* , in the Eastern Tropical Pacific.” Canadian Journal of Zoology 85: 108–121.

[ece374432-bib-0010] Evans, W. , J. Hall , A. Irvine , and J. Leatherwood . 1972. “Methods for Tagging Small Cetaceans.” Fishery Bulletin 70: 61–65.

[ece374432-bib-0011] Examiners Guide . 2021. “Marine Mammals Stranding Report‐Level A.” NOAA. Form 89‐864, OMB No. 0648‐0178.

[ece374432-bib-0012] Fair, P. , J. Adams , E. Zolman , et al. 2006. “Protocols for Conducting Dolphin Capture‐Release Health Assessment Studies.” NOAA. Technical Memorandum NOS NCCOS. pp. 49–83.

[ece374432-bib-0013] Fernandez, S. , and A. Hohn . 1998. “Age Structure, Growth, and Calving Seasonality of Bottlenose Dolphins Stranded in Texas.” Fishery Bulletin 96: 357–365.

[ece374432-bib-0014] Giordano, R. , H. Lyn , D. Suffoletta , et al. 2026. “Evaluating Movement Patterns of Common Bottlenose Dolphins Using Cookiecutter Shark Bite Wounds in the Mississippi Sound.” Journal of Cetacean Research and Management 27: 11–22.

[ece374432-bib-0015] Harkonen, T. , K. Harding , and S. Gunnar Lunneryd . 1999. “Age‐ and Sex‐Specific Behaviour in Harbour Seals *Phoca vitulina* Leads to Biased Estimates of Vital Population Parameters.” Journal of Applied Ecology 36: 825–841.

[ece374432-bib-0016] Hayes, S. , E. Josephson , K. Maze‐Foley , P. Rosel , J. McCordic , and J. Wallace . 2023. U.S. Atlantic and Gulf of Mexico Marine Mammal Stock Assessments 2022 (NOAA Technical Memorandum NMFS NE 304). National Oceanic and Atmospheric Administration, U.S. Department of Commerce.

[ece374432-bib-0017] Hohn, A. , and S. Fernandez . 1999. “Biases in Dolphin Age Structure due to Age Estimation Technique.” Marine Mammal Science 15: 1124–1132.

[ece374432-bib-0018] Hohn, A. , M. Scott , R. Wells , J. Sweeney , and A. Irvine . 1989. “Growth Layers in Teeth From Known Age, Free‐Ranging Bottlenose Dolphins.” Marine Mammal Science 5: 315–342.

[ece374432-bib-0019] Hohn, A. , L. Thomas , R. Carmichael , et al. 2017. “Assigning Stranded Bottlenose Dolphins to Source Stocks Using Stable Isotope Ratios Following the Deepwater Horizon Oil Spill.” Endangered Species Research 33: 235–252.

[ece374432-bib-0020] Hubard, C. , K. Maze‐Foley , K. Mullin , and W. Schroeder . 2004. “Seasonal Abundance and Site Fidelity of Bottlenose Dolphins ( *Tursiops truncatus* ) in Mississippi Sound.” Aquatic Mammals 30: 299–310.

[ece374432-bib-0021] Irvine, A. , and M. Scott . 1984. “Development and Use of Marking Techniques to Study Manatees in Florida.” Florida Scientist 47: 12–26.

[ece374432-bib-0023] McEntee, M. , V. Foroughirad , E. Krzyszcyk , and J. Mann . 2023. “Sex Bias in Mortality Risk Changes Over the Lifespan of Bottlenose Dolphins.” Proceedings of the Royal Society of London 290: 20230675. 10.1098/rspb.2023.0675 PMC10369037 37491966

[ece374432-bib-0024] Melin, S. , R. Ream , and T. Zeppelin . 2006. “Report of the Alaska Region and Alaska Fisheries Science Center Northern Fur Seal Tagging and Census Workshop.” 6–9 September 2005, Seattle, WA.

[ece374432-bib-0025] Miller, L. J. , A. D. Mackey , M. Solangi , and S. A. Kuczaj II . 2013. “Population Abundance and Habitat Utilization of Bottlenose Dolphins in the Mississippi Sound.” Aquatic Conservation: Marine and Freshwater Ecosystems 23: 145–151.

[ece374432-bib-0026] Mintzer, V. , A. Quackenbush , and K. Fazioli . 2023. “Site Fidelity of Common Bottlenose Dolphins ( *Tursiops truncatus* ) in a Highly Industrialized Area of the Galveston Bay Estuary.” Marine Mammal Science 39: 515–532.

[ece374432-bib-0027] Mullin, K. , T. McDonald , R. Wells , et al. 2017. “Density, Abundance, Survival, and Ranging Patterns of Common Bottlenose Dolphins ( *Tursiops truncatus* ) in Mississippi Sound Following the Deepwater Horizon Oil Spill.” PLoS One 12: e0186265. 10.1371/journal.pone.0186265 PMC5650146 29053728

[ece374432-bib-0028] Odell, A. , and E. Asper . 1982. “Live Capture, Marking, and Resighting of Bottlenose Dolphins. Final Report.” National Marine Fisheries. NA80‐GA‐C‐0041W.

[ece374432-bib-0029] Odell, D. , and A. Schneyer . 1983. “Age Estimation and Endocrinology in: Atlantic Bottlenose Dolphin, *Tursiops truncatus* , Herd Studies in the Mississippi Sound, USA: Capture, Freeze Marking and Biological Sampling. Final Report.” National Marine Fisheries. NA82 GA‐C‐0023.

[ece374432-bib-0030] Pamperin, K. , H. Lyn , D. Suffoletta , et al. 2025. “Staying Close to Home: Assessing Site Fidelity Patterns of Bottlenose Dolphins ( *Tursiops truncatus* ) in the Mississippi Sound.” Aquatic Mammals 51: 485–497.

[ece374432-bib-0031] Passadore, C. , L. Möller , F. Diaz‐Aguirre , and G. Parra . 2018. “High Site Fidelity and Restricted Ranging Patterns in Southern Australian Bottlenose Dolphins.” Ecology and Evolution 8: 242–256. 10.1002/ece3.3674 PMC5756869 29321867

[ece374432-bib-0032] Pitchford, J. , E. Pulis , E. Evans , J. Shelley , B. Serafin , and M. Solangi . 2016. “Seasonal Density Estimates of *Tursiops truncatus* (Bottlenose Dolphin) in the Mississippi Sound From 2011 to 2013.” Southeastern Naturalist 15: 188.

[ece374432-bib-0033] Rouby, E. , F. Plard , V. Ridoux , et al. 2025. “Longevity Collapse in Dolphins: A Growing Conservation Concern in the Bay of Biscay.” Conservation Letters 18: e13142.

[ece374432-bib-0034] Sherwin, R. , S. Haymond , D. Stricklan , and R. Olsen . 2002. “Freeze‐Branding to Permanently Mark Bats.” Wildlife Society Bulletin 30: 97–100.

[ece374432-bib-0035] Solangi, M. , and G. Dukes . 1983. Atlantic Bottlenose Dolphin, *Tursiops truncatus* , Herd Studies in the Mississippi Sound, U.S.A.: Capture, Freeze Marking and Biological Sampling. Final Report. National Marine Fisheries. NA82‐GA‐C‐0023.

[ece374432-bib-0036] Stacy, B. , B. Wallace , T. Brosnan , et al. 2019. “Guidelines for Oil Spill Response and Natural Resource Damage Assessment: Sea Turtles.” U.S. Department of Commerce, National Marine Fisheries Service and National Ocean Service, NOAA Technical Memorandum NMFS‐OPR‐61, 197 pp.

[ece374432-bib-0037] Suffoletta, D. , H. Lyn , L. Johnson , et al. 2026. “Sounding Out Reproduction: Bottlenose Dolphin ( *Tursiops truncatus* ) Calving Patterns in the Northern Gulf of Mexico.” Aquatic Mammals 52: 158–174.

[ece374432-bib-0038] Takeshita, R. , B. C. Balmer , F. Messina , et al. 2021. “High Site‐Fidelity in Common Bottlenose Dolphins Despite Low Salinity Exposure and Associated Indicators of Compromised Health.” PLoS One 16: e0258031. 10.1371/journal.pone.0258031 PMC8483354 34591903

[ece374432-bib-0039] Thompson, J. , V. Zero , L. Schwacke , et al. 2019. “finFindR: Computer‐Assisted Recognition and Identification of Bottlenose Dolphin Photos in R.” *bioRxiv*: the preprint server for biology.

[ece374432-bib-0040] Tschopp, A. , M. A. Ferrari , E. A. Crespo , and M. A. Coscarella . 2018. “Development of a Site Fidelity Index Based on Population Capture‐Recapture Data.” PeerJ 6: e4782. 10.7717/peerj.4782 PMC5949061 29761064

[ece374432-bib-0045] Vollmer, N. L. , P. E. Rosel , K. D. Mullin , et al. 2021. “Assessing Common Bottlenose Dolphin (*Tursiops truncatus*) Population Structure in Mississippi Sound and Coastal Waters of the North Central Gulf of Mexico.” Aquatic Conservation: Marine and Freshwater Ecosystems 31: 2951–2966.

[ece374432-bib-0041] Wells, R. , H. Rhinehart , L. Hansen , et al. 2004. “Bottlenose Dolphins as Marine Ecosystem Sentinels: Developing a Health Monitoring System.” EcoHealth 1: 246–254.

[ece374432-bib-0042] Wells, R. , and M. Scott . 1999. “The Bottlenose Dolphin.” In Handbook of Marine Mammals, Volume 6. The Second Book of Dolphins. Academic Press.

[ece374432-bib-0044] Wursig, B. , and S. Lynn . 1996. “Movements, Site Fidelity, and Respiration Patterns of Bottlenose Dolphins on the Central Texas Coast.” NOAA Technical Memorandum. NMFS‐SEFSC‐383.

